# Unlocking mRNA Vaccine Potential in Liver Cancer Treatment via Synergistic Bile Acid Modulation

**DOI:** 10.3390/vaccines13050502

**Published:** 2025-05-09

**Authors:** Yuqian Wang, Rui Han, Changquan Ling

**Affiliations:** 1 School of Integrative Medicine, Shanghai University of Traditional Chinese Medicine, Shanghai 201203, China; mek-nea@outlook.com; 2 Department of Traditional Chinese Medicine Oncology, The First Affiliated Hospital of Naval Medical University (Second Military Medical University), Shanghai 200433, China; 3 Faculty of Traditional Chinese Medicine, Naval Medical University (Second Military Medical University), Shanghai 200433, China

**Keywords:** bile acid, mRNA cancer vaccine, liver cancer, combinatorial therapeutic approach

## Abstract

This Letter to the Editor explores synergistic mechanisms enhancing mRNA cancer vaccine efficacy through bile acid metabolism modulation in liver cancer treatment. The latest evidence indicates that bile acids significantly impair T cell function within the liver cancer microenvironment, creating an immunosuppressive milieu that hampers anti-tumor responses. Modulating bile acid composition, particularly increasing ursodeoxycholic acid (UDCA), could reshape the tumor microenvironment (TME) to favor mRNA vaccine-induced T cell activity—a promising strategy to overcome current immunotherapy limitations in liver cancer.

The remarkable therapeutic potential of mRNA vaccines has revolutionized cancer immunotherapy approaches, especially in light of the compelling interim results from the pioneering KEYNOTE-942 trial [1]. For details, mRNA-4157 is an investigational personalized cancer vaccine that utilizes messenger ribonucleic acid (mRNA) technology, incorporating up to 34 synthetic mRNA molecules encoding neoantigens derived from patient-specific tumor DNA mutations [2]. This trial has demonstrated that mRNA-4157, in combination with pembrolizumab (anti-PD-1), reduced the risk of distant metastasis or death by approximately 65% compared to pembrolizumab monotherapy. Furthermore, the 12-month recurrence-free survival (RFS) rates were 83.4% (95% CI, 74.7–89.3) and 77.1% (95% CI, 62.5–86.6) for the combination and monotherapy groups, respectively, while the 18-month RFS rates were 78.6% (95% CI, 69.0–85.6) and 62.2% (95% CI, 46.9–74.3) [3]. While these results underscore the promise of personalized mRNA-based immunotherapy, their applicability to liver cancer is currently being explored. In a recent phase II clinical trial (NCT03067493), the personalized neoantigen mRNA vaccine LK101 was evaluated alongside standard ablative therapy in patients with hepatocellular carcinoma (HCC). The results were encouraging: all 12 vaccinated patients survived beyond four years while 3 control-group patients died during follow-up. The vaccine cohort exhibited substantially reduced recurrence rates at one year (18.2% vs. 33.3%) and two years (36.4% vs. 51.4%). LK101 maintained acceptable safety alongside ablative therapy while effectively inducing immune activation and potentially improving survival outcomes [4,5]. These findings provide preliminary evidence supporting mRNA vaccine application in HCC treatment. Multiple ongoing clinical investigations are further evaluating mRNA platforms for liver cancer [6], with results pending. These emerging investigations, together with preclinical mechanistic evidence, highlight the significant potential of mRNA vaccine platforms to transform liver cancer treatment.

The mechanism of action of mRNA cancer vaccines encompasses two critical components: antigen expression and immunomodulation. These vaccines can be engineered to express highly specific liver cancer antigens (such as GPC3, AFP, hTERT, and MAGE family proteins) that, upon cellular uptake, undergo processing and presentation via MHC molecules to activate CD8⁺ cytotoxic and CD4⁺ helper T lymphocytes, eliciting targeted anti-tumor responses. While unmodified mRNA activates pattern recognition receptors (TLR7/8, RIG-I) and induces interferon-mediated inflammation that potentially compromises translation, contemporary clinical mRNA vaccines incorporate nucleotide modifications (pseudouridine, N1-methylpseudouridine) that reduce innate immune recognition while enhancing protein expression [7]. Combined with lipid nanoparticle delivery systems, these modified mRNAs achieve an optimal therapeutic–safety balance—facilitating antigen presentation while preventing excessive inflammation that could disrupt tumor microenvironment (TME) homeostasis, thereby establishing durable anti-tumor immunity and potential recurrence protection [8]. This engineered “low immunogenicity-high translational efficiency” profile represents the fundamental advantage driving therapeutic promise of mRNA platforms in liver cancer immunotherapy.

However, as a typically immune-tolerant organ, the liver TME is enriched with various immunosuppressive components—such as regulatory T cells (Tregs), myeloid-derived suppressor cells (MDSCs), tumor-associated macrophages (TAMs), and immunosuppressive cytokines—which collectively impair T cell activation and limit the ability of mRNA vaccines to elicit robust anti-tumor responses [9]. Moreover, patients with liver cancer tend to exhibit reduced T-cell activation and generally impaired effector function and persistence, further compromising vaccine efficacy. Additionally, the high degree of antigenic heterogeneity in liver cancer facilitates immune escape, necessitating individualized or multi-antigen combination vaccine strategies to overcome this challenge. Current mRNA vaccines primarily rely on lipid nanoparticles (LNPs) or other delivery systems; however, vectors with high tumor-targeting capacity, low toxicity, and strong immunogenicity in liver cancers remain lacking. Notably, most patients with liver cancer have a background of cirrhosis, where vaccine-induced immune activation may exacerbate liver dysfunction. Although mRNA vaccines have shown therapeutic potential in various solid tumors, their clinical translation in liver cancer, including HCC and ICC, remains a significant challenge.

What is worth noting is that recent evidence suggests that modulating bile acid metabolism, particularly through the regulation of bile acid transporters and the elevation of certain bile acids, such as ursodeoxycholic acid (UDCA) [10], could potentially create a more favorable hepatic TME for mRNA vaccine-mediated immune responses. This approach offers a promising novel therapeutic breakthrough for enhancing the efficacy of current mRNA-based treatments. Bile acids are broadly categorized into primary (e.g., cholic acid [CA], chenodeoxycholic acid [CDCA]) and secondary bile acids (e.g., deoxycholic acid [DCA], lithocholic acid [LCA]), each possessing distinct immunomodulatory effects. For instance, hydrophilic bile acids such as UDCA exert anti-inflammatory and immunoprotective properties, whereas hydrophobic secondary bile acids including DCA and LCA have been associated with T cell dysfunction, endoplasmic reticulum stress, and oxidative damage within the TME [11]. Therefore, modulating bile acid metabolism, particularly elevating UDCA or altering the bile acid composition, may foster a more immune-supportive hepatic microenvironment conducive to mRNA vaccine efficacy. Additionally, bile acid accumulation within the TME has been implicated in promoting an immunosuppressive T cell phenotype, further restricting anti-tumor immune responses [10]. Studies indicate that decreasing bile acid aminotransferase (BAAT) activity or increasing UDCA levels can reduce the immunosuppressive bile acid concentration in tumor-associated tissues, thereby improving conditions for T cell function. UDCA, a naturally occurring bile acid, possesses anti-inflammatory and immunomodulatory properties that may enhance T cell infiltration and activity within tumors [12]. Thus, the combination of UDCA supplementation with mRNA vaccines may have a synergistic effect, mitigating local immunosuppression and fostering a robust T cell response.

mRNA cancer vaccines activate tumor-specific immunity through defined immunological cascades [8]. Dendritic cells (DCs) present antigen-MHC-I complexes to CD8⁺ cytotoxic T lymphocytes (CTLs) alongside CD80/86-CD28 co-stimulatory signals, while modified mRNA’s sustained antigen expression drives CTL differentiation into effector cells secreting perforin and granzymes [13]. Concurrently, CD4⁺ T-cells recognize antigen-MHC-II complexes and develop into Th1 cells producing IL-2, IL-12, and IFN-γ that amplify CTL function and recruit additional immune components. These vaccines combat immunosuppressive TME by enhancing tumor MHC-I expression while suppressing regulatory T-cells and M2 macrophages [14]. Importantly, while effective mRNA vaccines against infectious diseases often rely on both antibody and T cell responses, tumor immunity depends predominantly on Th1 and CTL responses. In contrast, antibody responses play a more limited role in directly eliminating solid tumors such as liver cancer [15]. Therefore, the T cell-mediated component of the immune response is considered critical for the success of mRNA-based cancer immunotherapies.

However, T cell dysfunction and exhaustion present significant challenges in cancer immunotherapy, particularly in liver cancer, where chronic antigenic stimulation can lead to impaired T cell responses [16]. Bile acid-associated endoplasmic reticulum stress and oxidative stress have been identified as key contributors to T cell dysfunction and exhaustion, with the liver being particularly susceptible due to its central role in bile acid metabolism [10]. Studies have shown that increasing the UDCA/LCA ratio reduces endoplasmic reticulum and oxidative stress, thereby improving T cell function, preserving their effector capacity [10], and enhancing mRNA vaccine-mediated anti-tumor immune responses. Furthermore, bile acid modulation may prevent early T cell exhaustion, thereby addressing a critical barrier in HCC treatment.

T cell survival and metabolic adaptability within the TME are essential for sustained anti-tumor immunity. Bile acid transporter proteins, such as *Slco3a1* and *Abcb1a/b*, play pivotal roles in enabling T cells to adapt to bile acid-rich environments by regulating bile acid homeostasis [10]. However, excessive bile acid accumulation disrupts T cell metabolic balance, compromising their survival and function. Research indicates that modifying bile acid metabolism or elevating UDCA levels enhances mitochondrial function, improves T cell metabolic fitness, and promotes their persistence in the TME [10]. Consequently, the combination of UDCA with mRNA vaccines holds promise for further augmenting T cell metabolic activity and anti-tumor efficacy.

In liver cancer, bile acids, which are abundant organ-specific metabolites, play a pivotal role in modulating immune responses. HCC tissues exhibit elevated levels of conjugated and secondary bile acids, which impair CD8⁺ T cell function via mechanisms involving oxidative stress and endoplasmic reticulum stress [10]. This immunosuppressive effect is primarily driven by intracellular accumulation of bile acids due to downregulation of bile acid efflux transporters in tumor-specific T cells. Additionally, the inhibition of bile acid synthesis through targeting *CYP7A1* or *CYP8B1* enzymes can significantly alter the bile acid pool, reducing immunosuppressive secondary bile acids while preserving primary bile acids that support T cell functionality. The selective modulation of bile acid transporters provides another therapeutic avenue to control bile acid distribution within the TME and prevent their accumulation in infiltrating lymphocytes. Modulation of bile acid metabolism, achieved via BAAT genetic ablation or UDCA supplementation, effectively mitigates metabolic competition between cancer cells and immune effectors, consequently potentiating antitumor immunity and restoring T cell functionality in experimental HCC models [10]. These interventions not only reduce tumor burden but also sensitize tumors to immune checkpoint blockade therapies such as anti-PD-1 antibodies. Given that mRNA cancer vaccines rely on the activation and persistence of cytotoxic T cells, integrating bile acid-modulating strategies may potentiate vaccine efficacy by overcoming metabolic immunosuppression in the liver TME. Therefore, rational combination strategies involving mRNA vaccines and metabolic interventions that normalize bile acid composition may offer synergistic benefits, enhancing tumor immunogenicity and T cell–mediated antitumor responses. This integrative approach may be particularly relevant in liver cancer, where organ-specific metabolic adaptations strongly influence therapeutic outcomes.

Moreover, building on the promising results of the KEYNOTE-942 trial, which combined immune checkpoint inhibitors (ICIs) with mRNA vaccines, the addition of bile acid modulators provides a compelling rationale for a triple-combination strategy [17]. This integrated approach–targeting metabolic immunosuppression through bile acid modulation, enhancing tumor antigen presentation via mRNA vaccination, and blocking inhibitory immune checkpoints–may elicit a comprehensive anti-tumor immune response capable of overcoming multiple layers of immune evasion in HCC.

The clinical translation of bile acid modulation as a synergistic strategy to enhance the efficacy of mRNA vaccines can be realized through several complementary approaches. Firstly, dietary interventions, such as increased intake or supplementation of UDCA during mRNA vaccine administration, represent a simple, cost-effective method to augment therapeutic outcomes by modulating the bile acid pool. Secondly, pharmacological strategies, including the development of BAAT inhibitors, offer a more targeted means of regulating bile acid metabolism, thereby enhancing vaccine-induced immune responses. Gene-therapy-based approaches may provide precise modulation of the TME by regulating bile acid biosynthesis or altering the expression of bile acid transporter proteins in T cells, ultimately improving T-cell activation and function within the TME. Furthermore, immune cell profiling offers mechanistic insights into how bile acid modulation shapes the intrahepatic immune landscape. Specifically, changes in key immune cell populations—such as BATF3⁺ DCs, CXCR3⁺ CD8⁺ T cells, and natural killer cells—could be assessed to evaluate alterations in the development and distribution of these immunologically relevant clusters following bile acid regulation. Notably, bile acid metabolism is closely linked to the gut microbiome, which modulates the differentiation and function of key immune subsets. One study demonstrated that gut-microbiome-regulated bile acid metabolites can shape hepatic natural killer T cells activity, thereby influencing anti-tumor immunity [18]. These findings suggest that clinical interventions involving oral bile acid modulation (e.g., UDCA supplementation) may have dual immunoregulatory effects—both directly within the liver and indirectly via gut microbiome—thereby strengthening the translational potential of this strategy. This approach underscores the potential of integrating bile acid modulation with mRNA vaccine strategies to reprogram the immunosuppressive TME and improve anti-tumor immunity in liver cancers. These approaches underscore the potential of integrating bile acid modulation with mRNA vaccine strategies to reprogram the immunosuppressive TME and improve anti-tumor immunity in liver cancers.

Of course, many related issues need urgent attention and exploration. These include, for example, evaluating the specific enhancing effects of UDCA on mRNA vaccines, both as monotherapy and in combination with ICIs; systematic assessment of safety issues, particularly regarding long-term bile acid modulation in liver immuno-oncology contexts; evaluating the synergistic effects of BAAT inhibition with various mRNA cancer vaccine platforms; and investigating specific bile acid metabolites as predictive biomarkers, which could help stratify patients and forecast therapeutic responses to mRNA-vaccine-based immunotherapy.

In conclusion, the bile-acid-based regulation strategy may offer a promising new avenue for enhancing the efficacy of mRNA cancer vaccines or even the therapeutic combination of mRNA vaccine plus ICI, in treating liver cancer, which have traditionally been less responsive to immunotherapy. Although further investigation and optimization are required, this approach warrants greater attention and research investment as a novel anti-tumor strategy.
